# The Effect of High Pressure Processing on Textural, Bioactive and Digestibility Properties of Cooked Kimberley Large Kabuli Chickpeas

**DOI:** 10.3389/fnut.2022.847877

**Published:** 2022-04-07

**Authors:** Prakhar Chatur, Stuart Johnson, Ranil Coorey, Rewati Raman Bhattarai, Sarita Jane Bennett

**Affiliations:** ^1^School of Molecular and Life Sciences, Curtin University, Bentley, WA, Australia; ^2^Ingredients by Design Pty Ltd., Perth, WA, Australia

**Keywords:** chickpeas, high pressure processing, texture, proximate composition, polyphenols, antioxidant activity, digestibility

## Abstract

High pressure processing is a non-thermal method for preservation of various foods while retaining nutritional value and can be utilized for the development of ready-to-eat products. This original research investigated the effects of high pressure processing for development of a ready-to eat chickpea product using Australian kabuli chickpeas. Three pressure levels (200, 400, and 600 MPA) and two treatment times (1 and 5 min) were selected to provide six distinct samples. When compared to the conventionally cooked chickpeas, high pressure processed chickpeas had a more desirable texture due to decrease in firmness, chewiness, and gumminess. The general nutrient composition and individual mineral content were not affected by high pressure processing, however, a significant increase in the slowly digestible starch from 50.53 to 60.92 g/100 g starch and a concomitant decrease in rapidly digestible starch (11.10–8.73 g/100 g starch) as well as resistant starch (50.53–30.35 g/100 g starch) content was observed. Increased starch digestibility due to high pressure processing was recorded, whereas *in vitro* protein digestibility was unaffected. Significant effects of high pressure processing on the polyphenol content and antioxidant activities (DPPH, ABTS and ORAC) were observed, with the sample treated at the highest pressure for the longest duration (600 MPa, 5 min) showing the lowest values. These findings suggest that high pressure processing could be utilized to produce a functional, ready to eat kabuli chickpea product with increased levels of beneficial slowly digestible starch.

## Introduction

High pressure processing (HPP) is a novel method of preserving foods, which is an alternative to standard heat treatment methods. It produces high quality and safe food which have a longer shelf life than conventionally stored food due to reduction of food spoiling microorganisms (1, 2). High pressure processing has been shown to retain quality attributes such as color, flavor and the nutritional value of foods (2, 3). In brief, during high pressure processing, at any given time, the pressure is transmitted uniformly by the pressure transmitting medium and is independent of the shape and size of the product being treated. The effectiveness of high pressure treatment depends on treatment time, pressure and temperature level, rate of pressurization/decompression, and on the composition of the food (4).

The demand for plant based protein is steadily growing around the world and is estimated to be a USD 14.5 billion market by 2025 (5). In the last 5 years increasing number of people shifted animal-based proteins to plant-based proteins sources in their meals (6, 7). Chickpeas (*Cicer arietinum* L.) are widely grown worldwide, are already a popular food source for humans and are an excellent source of proteins (8). However, the time required to cook chickpeas makes them a less popular choice amongst the younger generation, which prefers healthy and convenient food options requiring less preparation time (9).

As HPP is known to increase the shelf life of foods without affecting the sensory and nutritional properties (2), cooked and high pressure processed chickpeas could be introduced as a ready-to-eat option, with desirable textural, organoleptic and nutritional properties. However, there is a scarcity of knowledge on the effects of HPP on the textural, nutritional and bioactive properties of legumes in general. HPP denatures proteins reversibly and irreversibly. A moderate pressure (<300 MPa) affects the acceleration of enzyme action whereas, a pressure above 300 MPa induces protein denaturation and inactivation of enzymes (3, 10). Previous studies have reported the effect of high pressure on lentil starch (11), mung bean starch (12), and pea starch (13), however, no information is available on both protein and starch digestibility of HPP chickpeas.

A small decrease in the total polyphenols (9) and antioxidant properties of HPP vegetables have been reported by Butz et al. (14) whereas Doblado et al. (15) reported slight changes in the antioxidant capacity and vitamin C content of HPP germinated cowpeas [*Vigna unguiculata* (L.)], a grain legume common in sub-Saharan Africa, following high pressure treatment. A recent study by Alsalman and Ramaswamy (16) reported changes in the texture, color and antinutrient (tannins and phytic acid) content of raw Canadian kabuli chickpeas, but did not explore the changes in starch or protein digestibility, polyphenol content and antioxidant capacities of cooked chickpeas. Therefore, there is a need to investigate the effect of high pressure processing on the texture profile, general nutrient composition, starch and protein digestibility, polyphenol content and antioxidant capacity of cooked and HPP kabuli chickpeas, compared to cooked only samples to inform ready to eat product development in the near future. To fill this knowledge gap, the objective of this research work was to examine the effects of HPP (200, 400, and 600 MPa for 1 and 5 min) on the textural, nutritional, and bioactive properties of cooked Australian kabuli chickpeas.

## Materials and Methods

Representative samples of the cultivar Kimberley Large kabuli chickpeas (17) harvested in 2017 from the Ord River region of Western Australia were collected, sieved, vacuum packaged and stored at 4°C until analysis. The kabuli chickpeas were soaked in excess water overnight (12 h) at room temperature (22°C). After 12 h, the remaining water was drained off and chickpeas were cooked in fresh boiling water on a gas stove for 30 min as the seeds still maintained their shape and firm texture. Cooking for a longer duration resulted in seed coat separation. Following cooking, chickpeas were cooled by washing them under running tap water and excess moisture was removed using paper towel. The cooked and cooled chickpeas were vacuum packaged in a 100 micron vacuum packaging bag using a Multivac double chamber vacuum packaging machine (Model- C450, Multivac, Keilor Park, Victoria, Australia) and stored at 4°C until high pressure processing.

### High Pressure Processing

Cooked and vacuum packaged Kimberley Large kabuli chickpeas were commercially high pressure processed at Preshafoods Pty. Ltd. (Derrimut, Victoria, Australia) using hyperbaric high pressure processing equipment (Model- Hyperbaric 300, Burgos, Spain, EU) at the following settings detailed in Table 1.

**TABLE 1:** Pressure and time combinations for high pressure processing of cultivar Kimberley Large chickpea samples.

Time/Pressure	200 MPa	400 MPa	600 MPa
1 min	200 MPa, 1 min (2K1)	400 MPa, 1 min (4K1)	600 MPa, 1 min (6K1)
5 min	200 MPa, 5 min (2K5)	400 MPa, 5 min (4K5)	600 MPa, 5 min (6K5)

The time required to reach 200, 400, and 600 MPa was 60, 130, and 240 s, respectively, and the decompression was instant. Purified water was used as the pressurization media and all the samples were pressurized at 4°C. After processing, the samples were stored at 4°C and transported to Curtin University (Bentley, Western Australia) under refrigerated conditions. Cooked and vacuum packaged Kimberley Large kabuli chickpeas which were not subjected to high pressure processing were used as a control.

### Texture Profile Analysis

Texture profile analysis of the control and high pressure processed chickpeas was conducted using a Perten Texture analyzer (TVT6700, Hägersten, Sweden) (18). The samples were subjected to 50% compression with a perspex cylindrical probe (25 mm diameter) at a crosshead speed of 1 mm/s twice in two cycles using a 5 kg load cell. The texture profile was expressed as firmness, cohesiveness, springiness, gumminess and chewiness. Forty replicates were analyzed for control and each HPP sample.

### Moisture Content

Moisture content was determined in duplicate by the modified solids-(total) and moisture in flour-oven drying method (Method 925.10, (19).

### Protein Content

Protein content was determined in duplicate by Kjeldahl assay: digestion, distillation and titration using a nitrogen conversion factor of 6.25 (Method 920.87) (20).

### Total Starch Content

Starch content was determined in duplicate using the standard colorimetric technique (Method 996.11) (21), using the total starch assay kit (AA/AMG) from Megazyme International Ltd (Bray, County Wicklow, Ireland).

### Freeze Drying

The chickpea samples were placed in a freezer over night at –20°C. The samples were transferred to a freeze dryer (Christ Alpha 1-4LD Plus, Germany) and freeze-dried at 1 mbar pressure and –50°C for 4 days. The freeze-dried samples were milled (Grindomix, GM200, Retsch, Haan, Germany) to get a fine powder, passing 100% through 500 microns.

### Mineral Content

Inductively coupled plasma optical emission spectrometry (ICP-OES) (Varian, Palo Alto, United States) was used to determine the content of individual minerals (mg) in duplicate at the National Measurement Institute, Perth, WA. Freeze-dried control and HPP samples (1 g) were digested at 95°C for 2 h in a DEENA automated digestion block (Thomas Cain, Omaha, Nebraska, United States), after addition of concentrated nitric acid (HNO_3_) (3 mL) and concentrated HCl (3 mL). After digestion, distilled water was used to make the sample volume up to 40 mL, and the solutions were left to settle. Afterward, all the samples were diluted fivefolds with distilled water before being analyzed using ICP-OES. Appropriate emission wavelengths (higher sensitivity and lower interferences) of 238.204, 317.933, 213.618, 213.857, 279.078, 766.491, and 589.592 nm were chosen to analyze Fe, Ca, P, Zn, Mg, K, and Na, respectively (22). The mineral content was expressed as mg/kg sample.

### Polyphenol Extraction and Total Polyphenol Content

To extract total polyphenols, freeze-dried control and HPP samples (1 g) were mixed with 10 mL 50% acetone solution. The mixture was shaken at ambient temperature at 60 rpm for 3 (h) using a suspension mixer (RSM7DC, Ratek, Boronia, Victoria, Australia). The mixture was extracted for an additional 12–16 h in the dark overnight. The extract was centrifuged using an Eppendorf centrifuge (Model—5810r, Hamburg, Germany) at 12,000 *g*, for 10 min at room temperature, and the extracts were kept in the dark at −20°C until use (23).

Total polyphenol content was determined using Folin-Ciocalteu method (24). In brief, 100 μL of polyphenol extracts were mixed with 2.5 mL of 0.2 N Folin-Ciocalteu reagent, followed by 2 mL of saturated sodium carbonate solution (75 g/L) addition. After a reaction time of 2 h at room temperature in the dark, the absorbance of obtained mixtures was determined using a UV-1800 Spectrophotometer (Shimadzu, Canby, United States) at 765 nm. A standard curve was prepared using Gallic acid (0–360 mg/L) and results were expressed as mg Gallic acid equivalents (GAE)/100 g dry basis (db). All extract were analyzed in duplicate.

### Antioxidant Capacity

For the 2, 2-diphenyl-1-picrylhydrazyl (DPPH) assay, firstly, DPPH (24 mg) was added into 100 mL methanol to prepare the stock solution, which was stored at –20°C in the dark until use. To prepare the working solution, a 10 mL stock solution was diluted with 50 mL methanol, giving an absorbance of 1.1 ± 0.02 units at 515 nm. Duplicate kabuli chickpea phenolic extracts (150 μL) were mixed with the DPPH working solution (2,850 μL) and reacted for 2 h in the dark, after which the absorbance was determined at 515 nm and results were expressed in terms of mg Trolox equivalents (TE)/g db. Different concentrations of trolox (20–250 mg/L) were used to prepare a standard curve for DPPH antioxidant activity determination.

For the 3-ethylbenzothiazoline-6-sulfonic acid diammonium salt (ABTS) assay, a fresh stock solution was prepared by mixing 7.4 mM ABTS and 2.6 mM potassium per sulfate in equal amounts, which were kept for the duration of 12 h in the dark. Using the stock solution, a fresh ABTS working solution was prepared by diluting 1 mL of the stock solution with 60 mL of methanol to get a 1.1 ± 0.02 units absorbance at 734 nm. The kabuli chickpea phenolic extract (150 μL) was mixed with the ABTS working solution (2,850 μL) and incubated in the dark for 2 h, followed by absorbance determination at 734 nm. The standard used for the ABTS assay was Trolox (20–250 mg/L) and the results were expressed in terms of mg Trolox equivalents (TE)/g db, with all extracts being analyzed in duplicate (25). The oxygen radical absorbance capacity (ORAC) assay was performed using a modified method (26). All extracts were analyzed in duplicate and the final antioxidant activity results were calculated using Trolox as a standard (0–50 μmol/L), and expressed as μmol TE/100 g sample, db.

### Starch Isolation

Starch from the freeze-dried control and high pressure processed chickpea samples was isolated using the method of Sun et al. (27). Freeze-dried high pressure processed chickpea sample (250 g) was steeped in water containing 0.1% sodium sulfite for 12 h at 25°C. The slurry obtained was then diluted with 100 mL distilled water, and the pH was adjusted to pH 10 using 0.5 M sodium hydroxide (NaOH). The slurry was continuously mixed using a magnetic stirrer (500 rpm) for 1 h and then filtered through a 100 mesh sieve (0.149 mm nominal sieve opening) to separate the fiber from the starch. The filtered slurry was centrifuged at 3,000 *g* for 10 min at 10°C (Eppendorf Centrifuge 5810R made in Germany) and the supernatant was discarded. The centrifugation step was repeated six times until a clear supernatant was obtained whereas, pure starch was the white sediment remaining at the bottom. The isolated starch was dried using a digital hot air oven (Thermotec 200, Contherm, Lower Hutt, New Zealand) at 40°C for 48 h and ground into a fine powder using a pestle and mortar.

### Scanning Electron Microscopy

Isolated starch sample morphology was investigated using Tescan Mira3 FESEM with Oxford Instruments X-MaxN 150 silicon drift X-ray detector and Aztec software. Starch samples were fixed on a circular metallic microscope stub with carbon aluminum tape and then coated with a 5 nm platinum coating using a sputter coater (208HR, Cressington). The scanning electron microscopy was performed at an accelerating voltage of 2 kV.

### Attenuated Total Reflectance Fourier Transform Infrared Spectra Analysis

Attenuated total reflectance Fourier transform infrared spectra (ATR-FTIR) were collected using a Nicolet IFS50 FTIR spectrometer, and a single bounce diamond ATR accessory. Spectra were recorded by placing the milled freeze-dried sample in contact with the ATR accessory. Spectra were recorded across the spectral range 4,000–400 cm^–1^ at 4 cm^–1^ spectral resolution, with 128 co-added scans. The background spectrum was collected from blank diamond ATR crystals. Spectra were post normalized using vector normalization across the spectra range 875–1,190 cm^–1^, using OPUS software (v7.0). Second-derivatives of the FTIR spectra were then calculated using a 17-smoothing point Savitzky-Golay function.

### *In vitro* Slowly Digested Starch

*In vitro* slowly digested starch was determined in duplicate on fresh sample by a modified rapid glucometer method (28). A 250 mg weighed sample was placed in a 150 mL glass jar (Ergo Flint Glass 70 mm IM-106PK-1432-FL12, Plasdene Glass-Pak, Canning Vale, Australia) to which 1 mL of porcine α-amylase (250 U/mL in 0.2 M pH 7 carbonate buffer) was added. Twenty seconds later, 5 mL pepsin suspension [9 mg of pepsin (2,500 units/mg) and 5 mL 0.02 molar hydrochloric acid] was added that lowered the pH to that approximating *in vivo* gastric conditions (pH 2.0). The mixture was incubated at 37°C in a reticulating water bath (85 rpm) for 30 min prior to neutralization with 5 mL 0.02 M NaOH and 50 mL 0.2 M pH 6 sodium acetate buffer. This was followed by 5 mL pancreatin mixture [0.095 g pancreatin (Chem-Supply PL378) added to 3.325 mL Diaxame and 44.175 mL 0.2 M acetate buffer (pH 6.0)]. Incubation continued at 37°C in a reticulating water bath (85 rpm). Duplicate glucose readings were made at 0, 5, 10, 20, 40, 60, 90, and 120 min from the time of addition of the pancreatin mixture using an Accu Check Performa glucometer (Roche Diagnostics Aust. Pty. Ltd, Castle Hill, Australia). Slowly digested starch (SDS) in g per 100 g dry starch was calculated using Eq. (1).


(1) $$\text{SDS}=\frac{0.9\times \left(\mathrm{G120}-\text{G}\,20\right)\times 180\times \text{V}}{\text{W}\times S\,\left(100-M\right)}$$

where, G120 = glucometer reading (mM) at 120 min, G20 = glucometer reading (mM) at 20 min, V = volume of digesta (mL), 180 = molecular weight of glucose, W = weight of sample (g), S = starch content of sample (g per 100 g dry sample), M = moisture content of sample (g per 100 g sample), and 0.9 = stoichiometric constant for starch from glucose contents. The glucometer was corrected using a calibration curve with known concentrations of glucose in digesta at 37°C measured in duplicate with the glucometer. The glucometer readings were corrected for the readings at time zero which represented the free glucose present in the enzyme preparations used in the study.

### Protein Digestibility

*In-vitro* protein digestibility of control and HPP sample (6K5) was determined following a pepsin digestion method (29) with a few modifications. Approximately, 100 mg freeze-dried chickpea sample were incubated at 37°C with 0.75 mg pepsin (2,500 units/mg activity; Chem-Supply, Gillman, SA, Australia) in 7.5 ml of 0.1 N hydrochloric acid (HCl) for 5 h. The solution was then neutralized with 3.75 ml of 0.2 N NaOH. The undigested protein in 15 ml of digesta was then precipitated by addition of 25 ml of 10% tricholoroacetic acid and the sample centrifuged for 30 min at 2,000 × *g* at room temperature (22°C). Nitrogen in the supernatant was determined using the Kjeldahl digestion and distillation method. The *in-vitro* protein digestibility (IVPD) was calculated according to Eq. (2).


(2) $$\mathrm{IVPD}\left(\%\right)=\frac{\begin{array}{c} (\mathrm{Total}\mathrm{nitrogen}\left(g/100\mathrm{mL}\right)\\ -\mathrm{nitrogen}\mathrm{in}\mathrm{supernatant}\left(g/100\mathrm{mL}\right)) \end{array}}{\mathrm{Total}\,\mathrm{nitrogen}\,\left(g/100\,\mathrm{mL}\right)}\times 100$$

### Statistical Analysis

Two-way analysis of variance (ANOVA) was used to compare means of replicate analyzes per treatment followed by Duncan’s multiple range test to separate means when F was significant (<0.05). Dunnett’s *post-hoc* test was used to compare mean values of the control against those of high pressure treated samples. All tests were performed using Genstat statistical tool (Version- V.20.1.2.24528, VSN International Ltd., United Kingdom).

## Results and Discussion

### Texture Profile Analysis

**FIGURE 1:**
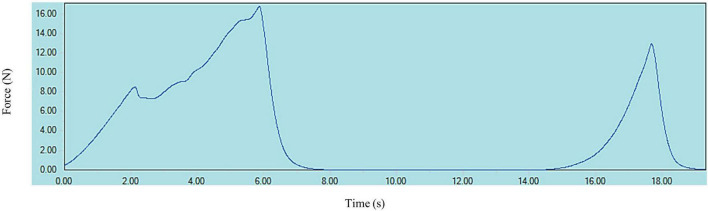
A typical texture profile curve for cooked kabuli chickpeas.

**FIGURE 2:**
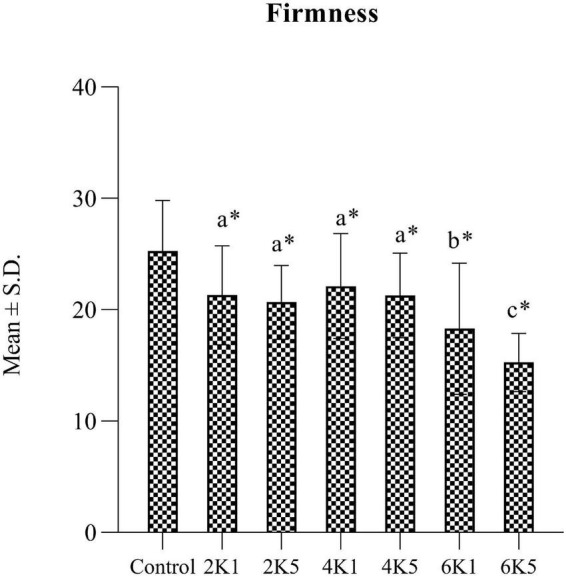
Firmness (N) of control and HPP chickpea samples (2K1 = 200 MPa, 1 min; 2K5 = 200 MPa, 5 min; 4K1 = 400 MPa, 1 min; 4K5 = 400 MPa, 5 min; 6k1 = 600 MPa, 1 min; and 6K5 = 600 MPa, 5 min). Bars bearing different letter are significantly (*p* < 0.05) different to each other. Bars bearing * are significantly (*p* < 0.05) different to the control.

**FIGURE 3:**
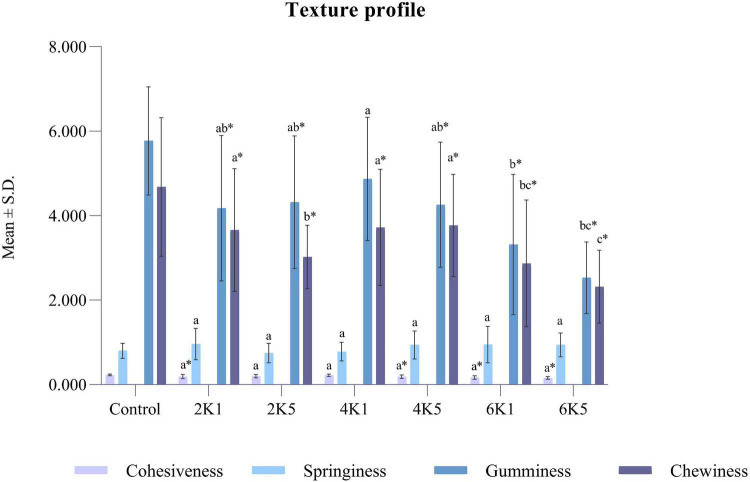
Texture profile of control and HPP chickpea samples (where, 2K1 = 200 MPa, 1 min; 2K5 = 200 MPa, 5 min; 4K1 = 400 MPa, 1 min; 4K5 = 400 MPa, 5 min; 6k1 = 600 MPa, 1 min; and, 6K5 = 600 MPa, 5 min). Bars bearing different letter for the same parameter are significantly (*p* < 0.05) different. Bars bearing * for the same parameter are significantly (*p* < 0.05) different to the control.

A typical texture profile analysis (TPA) curve for cooked kabuli chickpea samples is shown in Figure 1. Texture measurements of control and HPP samples are presented in Figures 2, 3, where high pressure processing resulted in significant (*p* < 0.05) changes in TPA parameters when compared to the control following ANOVA. In addition, the 5 min application at all pressures lead to a significant (*p* < 0.05) reduction in TPA values than samples treated for 1 min. The firmness of samples ranged from 15.27 to 25.28 N and was highest for the control and lowest for 6K5. Two-way analysis of variance (ANOVA) showed a significant effect (*P* < 0.05) of pressure (P) on firmness of samples, however, no effect of time (T) or pressure and time (P x T) interaction was observed. Samples treated at 200 and 400 MPa had similar firmness values, however, with the increase in treatment pressure to 600 MPa, a significant reduction in firmness of samples was observed. When compared to the control, even the lowest processing treatment (2K1) significantly (*p* < 0.05) reduced the firmness of cooked chickpeas and all high pressure treated samples were significantly (*p* < 0.05) less firm than the control, with 6K5 being the softest. Alsalman and Ramaswamy (16) also reported a decrease in firmness of chickpeas with increase in pressure (from 100 to 400 MPa). However, an increase in pressure to 600 MPa led to an increase in firmness, possibly due to aggregation of protein in the uncooked samples used in this study (30). Tissue collapse, weakened hydrophobic interactions of protein matrix and internal redistribution of moisture can be attributed to texture degradation (31), resulting in loss of firmness.

Cohesiveness in the texture profile analysis indicates how well a product withstands a second deformation following the first. Cohesiveness for cooked and chickpeas ranged between 0.16 and 0.23 and was lowest for 6K5 and highest for the control. Cohesiveness can be influenced by the depressurization process and hence a more pronounced effect can be observed for samples treated at 600 MPa. Springiness, gumminess, and chewiness for the cooked and samples ranged between 0.80 and 0.96 mm, 2.53–5.77 N, and 2.32–4.68 N mm, respectively. Similar to the firmness, cohesiveness, gumminess and chewiness of treated samples were significantly (*p* < 0.05) affected by the pressure treatment (P), however, there was no effect of time (T) or P x T interaction on these texture properties. Cohesiveness values for 2K1, 4K5, 6K1, and 6K5 were significantly (*P* < 0.05) lower than the control. Also, all samples were significantly (*P* < 0.05) less gummy (except 4K1) and less chewy than the control. Springiness is a degree to which cooked grains can return to their original shape after partial compression. Springiness negatively correlates with hardness and chewiness, suggesting that springier samples are less hard and less chewy (32). No effect of high pressure processing on springiness of cooked kabuli chickpeas was observed in this study.

The effect of high pressure on firmness, cohesiveness, springiness, gumminess, and chewiness has been investigated in previous studies on the texture of beetroot (33), meat (30, 34) and chickpeas (16). However, due to the conflicting results from these studies, a conclusion based on the available data to support the clear effects of high pressure processing on these textural properties cannot be reached. Our result for firmness was in agreement with that of Koca et al. (31) for cheese, Yu et al. (35) for brown rice and Alsalman and Ramaswamy (16) for chickpeas, which also reported that increasing pressure during HPP results in reduction in sample firmness.

In this study, softest texture was chosen as desirable based on the results of past sensory studies on legumes such as bengal gram (*Cicer arietinum*), green gram (*Phaseolus aureus Roxb*), lentils (*Lens esculenta*) (36), and beans (*Phaseolus vulgaris*) (37). Among all the samples, 6K5 showed the lowest firmness, cohesiveness, gumminess and chewiness values, whereas the control had the highest values for these textural properties. Thus, based on instrumental analysis we can concur that 6K5 had the most desirable textural properties out of all the samples in this study.

### General Nutrient Composition

The proximate and dietary fiber content of the control and HPP chickpea samples is given in Table 2. The moisture content of the HPP samples ranged from 61.01 to 64.13 g/100 g, and the protein content ranged from 17.8 to 19.02 g/100 g db. None of these measurements were significantly different to the control. On the other hand, the starch content of the HPP samples ranged from 46.13 to 54.97 g/100 g and was significantly (*P* < 0.05) affected by treatment pressure levels, with an increase in pressure, leading to a decrease in starch content. When compared to the control, 4K1, 4K5, 6K1, and 6K5 had significantly (*P* < 0.05) less starch content. The reason for reduction in starch content is leaching of starch due to rupture of cell walls by the high pressure applied as observed in common beans as well (38). Based on these results, HPP can be potentially used to reduce starch content of other high starch grains and pulses for designing lower calorie alternatives.

**TABLE 2:** Moisture, protein, starch and dietary fiber (soluble, insoluble, and total dietary fiber) content of control and high pressure processed cooked chickpeas.

Component (g/100g)	Control	200 MPa, 1 min	200 MPa, 5 min	400 MPa, 1 min	400 MPa, 5 min	600 MPa, 1 min	600 MPa, 5 min
Moisture	62.65 ± 1.30	61.01 ± 1.46^a^	62.53 ± 3.40^a^	62.60 ± 1.49^a^	61.44 ± 1.51^a^	64.13 ± 1.41^a^	62.62 ± 1.23^a^
Protein	17.09 ± 1.21	17.81 ± 1.43^a^	17.94 ± 1.24^a^	19.02 ± 1.57^a^	17.44 ± 0.17^a^	18.65 ± 0.78^a^	17.96 ± 2.12^a^
Starch	54.48 ± 2.18	54.97 ± 2.26^a^	53.86 ± 1.30^ab^	49.46 ± 4.57^bc^*	47.41 ± 1.99^c^*	47.29 ± 1.26^c^*	46.13 ± 0.59^c^*
**Dietary fiber**							
SDF	2.408 ± 0.03	2.244 ± 0.39^a^	2.817 ± 0.18^a^	2.267 ± 0.10^a^	3.094 ± 0.28^a^	2.412 ± 0.49^a^	3.189 ± 0.06^a^*
IDF	20.951 ± 0.30	21.417 ± 0.56^a^	18.571 ± 0.68^b^	20.373 ± 0.07^ab^	20.026 ± 0.61^ab^	20.414 ± 0.16^ab^	19.409 ± 0.39^ab^
Total	23.359 ± 0.33	23.662 ± 0.96^a^	21.388 ± 0.50^a^	22.64 ± 0.02^a^	23.12 ± 0.88^a^	22.826 ± 0.65^a^	22.598 ± 0.33^a^

SDF, soluble dietary fiber; IDF, insoluble dietary fiber. All values are presented as mean ± standard deviation. Following Duncan’s post hoc test, values followed by the same superscript within a row do not differ significantly (p < 0.05). *Denotes significant difference (p < 0.05) between control and HPP sample following Dunnett’s test.

The soluble dietary fiber (SDF), insoluble dietary fiber, and total dietary fiber content ranged from 2.244 to 3.189 g/100 g db, 18.571 to 21.417 g/100 g db, and 21.388 to 23.662 g/100 g db, respectively. Treatment time (T) had a significant (P < 0.05) effect on the SDF content of the HPP samples, with the increase in treatment time from 1 to 5 min, resulting in a marked increase in SDF levels. Whereas pressure x time (P x T) interaction had a significant (*p* < 0.05) negative effect on the IDF and total dietary fiber (TDF) levels. With an increase in treatment pressure (from 200 to 600 MPa) and time (1–5 min), the IDF and TDF levels decreased markedly. However, when compared to the control, all the samples had similar SDF, IDF and TDF levels, with only 6K5 having a significantly (*P* < 0.05) higher SDF content. The significant increase in SDF content of sample 6K5 and the marked reduction in IDF and TDF levels of all samples can be a result of cleavage of glycosidic linkages and/or breakage of weak bonds between polysaccharides (39). Consumption of SDF has shown to reduce postprandial glucose absorption in humans, acting as a glycemic lowering ingredient. Thus, HPP can be used in further research and development of legume based functional foods.

### Mineral Content

The mineral content of the control and HPP sample (6K5) is reported in Table 3. Potassium was the most abundant mineral (5,371–6,131 mg/kg), followed by phosphorus (3,749–4,173 mg/kg), magnesium (1,267–1,340 mg/kg) and calcium (917–994 mg/kg). Other studies have reported that compared to their raw counterparts, soaked and/or cooked legumes have a reduced mineral content (40–42). The cooked samples from this study demonstrated higher iron, phosphorus, and magnesium levels when compared to uncooked chickpea samples from other region of the world possibly due to solid loss during cooking (43), however, the effect of high pressure processing on the mineral content of cooked kabuli chickpeas has previously not been reported.

**TABLE 3:** *In vitro* Protein digestibility and mineral content of the control and high pressure processed cooked chickpeas.

Parameters	Control	600 MPa, 5 min
IVPD (%)	78.078 ± 1.25^a^	79.046 ± 3.29^a^
**Minerals (mg/kg)**		
Calcium	994 ± 59^a^	917 ± 10^a^
Iron	62 ± 4.9^a^	59 ± 1.4^a^
Potassium	6,131 ± 416^a^	5,371 ± 18^a^
Magnesium	1,340 ± 157^a^	1,267 ± 76^a^
Sodium	75 ± 4.9^a^	74 ± 1.4^a^
Phosphorus	4,173 ± 108^a^	3,749 ± 12^a^
Zinc	42 ± 4.2^a^	40 ± 0.7^a^

*IVPD, In vitro protein digestibility. All values are presented as mean ± standard deviation. Following Duncan’s post hoc test, values followed by the same superscript within a row do not differ significantly (p < 0.05).*

High hydrostatic pressure processing is known to not affect smaller molecules such as volatile compounds, pigments, vitamins and compounds related to sensory, nutritional and health promoting effects (3, 44). In our study, no significant (*P* < 0.05) differences were observed in the mineral content of the control and the highest treatment level sample, meaning when consumers ingest HPP chickpeas, they receive the same total intake of minerals as with traditionally cooked chickpeas. Our results are also in agreement with studies on other foods treated with HPP such as Andrés et al. (45) who reported no effect of HPP on the mineral content of milk and soy smoothies containing fruits (orange, papaya, melon and carrot). As no difference between the control and highest treatment level sample was found, samples with lower HPP treatments were not analyzed for their mineral content.

### Polyphenol Content

The polyphenol content of the control and HPP samples is reported in Table 4. It ranged from 31.704 to 52.321 mg gallic acid equivalent/100 g sample (d.b.), with the highest recorded for 2K1 and the lowest for 6K5. Main and significant (*P* < 0.05) effects of pressure and treatment time were observed, suggesting that with an increase in pressure and treatment time, total polyphenol content (TPC) in samples decreased. However, when compared to the control, only samples treated at 600 MPa were significantly (*P* < 0.05) lower in their TPC.

**TABLE 4:** Polyphenol content and antioxidant capacity of control and high pressure processed cooked chickpeas.

Parameters	Control	200 MPa, 1 min	200 MPa, 5 min	400 MPa, 1 min	400 MPa, 5 min	600 MPa, 1 min	600 MPa, 5 min
TPC (mg GAE/100 g)	49.390 ± 1.05	52.321 ± 3.16^a^	51.478 ± 1.56^a^	51.341 ± 1.81^a^	49.712 ± 1.53^a^	34.159 ± 1.61^b^*	31.704 ± 1.01^b^*
DPPH (mg TE/100 g)	55.511 ± 0.92	58.480 ± 1.50^a^	56.542 ± 0.61^a^	48.731 ± 2.81^b^*	46.351 ± 1.79^b^*	33.693 ± 2.02^c^*	29.722 ± 1.97^c^*
ABTS (mg TE/100 g)	91.050 ± 2.09	91.022 ± 3.38^a^	90.309 ± 0.89^a^	90.783 ± 1.07^a^	90.678 ± 1.34^a^	89.813 ± 1.46^a^	85.506 ± 0.82^b^*
ORAC (μ mol TE/100 g)	1,996 ± 72	1,993 ± 97^a^	1,875 ± 224^a^	2,054 ± 267^a^	1,937 ± 128^a^	1,723 ± 196^a^	1,253 ± 233^b^*

TPC, total polyphenol content; DPPH, 2-2-diphenyl-1-picrylhydrazyl antioxidant assay; ABTS, 3-ethylbenzothiazoline-6-sulfonic acid diammonium salt antioxidant assay; ORAC, oxygen radical antioxidant capacity. All values are presented as mean ± standard deviation. Following Duncan’s post hoc test, values followed by the same superscript within a row do not differ significantly (p < 0.05). *Denotes significant difference (p < 0.05) between control and HPP samples following Dunnett’s test.

Similar results have been reported by Rodríguez-Roque et al. (46) and Barba et al. (47), who also found a slight decrease in the polyphenol content of orange juice following high pressure treatment above 400 MPa. Linsberger-Martin et al. (9) also reported a reduction in the total phenolic content in split peas and whole white beans after HPP. In contrast, Wang et al. (48), Sánchez-Moreno et al. (49), Patras et al. (50), and Patras et al. (51) reported an increase in the polyphenol content of different plant foods (cereals, tomato puree, tomato, carrot, strawberry, and blueberry, respectively) after high pressure processing.

The type of food, location of phenolic compounds in food, pre-processing treatment, as well as the duration and intensity of the HPP treatment affects the concentration of phenolic compounds in the extract (52). An increase in the total phenolic content of certain foods (in case certain fruits and vegetables) can be due to the disruption of cell wall structures, inactivation of enzymes related to loss of phenolic substances or improved extractability of the antioxidant components following high pressure treatment (47). However, changes in the physicochemical characteristics (46) and enhanced chemical and enzymatic oxidation of polyphenols (53) can occur due to processing, resulting in a lower availability.

### Antioxidant Capacity

An effect of high pressure processing on the antioxidant capacity of cooked kabuli chickpeas was observed and the values are reported in Table 4. The DPPH, ABTS and ORAC values for HPP samples ranged from 29.722 to 58.480 mg trolox equivalent/100 g, 85.506–91.022 mg TE/100 g, and 1,253–1,993 μmol TE/100 g, respectively. For DPPH and ORAC antioxidant capacity, significant (*P* < 0.05) negative effects of pressure and time were observed. Thus, with an increase in pressure (200–600 MPa) and treatment time (1–5 min), the DPPH and ORAC values significantly decreased. However, the reduction in ORAC values was only observed at the highest pressure level (600 MPa). Significant (*P* < 0.05) main effects of pressure (P), time (T) and PxT interaction were observed for ABTS antioxidant capacity. However, following Duncan’s *post hoc* test, only sample 6K5 showed significantly (*P* < 0.05) lower ABTS value when compared to all other samples including control. When compared to the control, 4K1, 4K5 6K1, and 6K5 showed significantly (*P* < 0.05) lower DPPH antioxidant values, whereas only 6K5 expressed significantly (*p* < 0.05) lower ABTS and ORAC value than the control. Sample 6K5 had the overall highest decrease in the antioxidant capacities out of all samples analyzed.

The DPPH and ABTS assays are considered more accurate and reliable when compared to other methods such as FRAP (Ferric reducing antioxidant power) because of their rapidity, robustness and reliability in assessing antioxidant capacity of plant materials (54). On the other hand, ORAC assay uses biologically relevant free radicals, is standardized and integrates both degree and time of antioxidant reaction unlike other chemical assays (55). Previous studies have recommended to use at least 2 assays for the antioxidant capacity analysis of plant materials (54, 56), and thus we have chosen these three assays for this study to validate our results.

Negative effects of thermal treatments on the antioxidant capacity of legumes such as chickpeas, soybeans, kidney beans (57), common beans (58), and cowpeas (59) have been reported in previous studies. However, very limited information on the effects of high pressure processing on the antioxidant capacity of legumes or foods in general is available. Doblado et al. (15) observed a significant decrease in the ABTS antioxidant capacity of germinated cowpeas after HPP of up to 500 MPa (room temperature/15 min), and Butz et al. (14) reported an 11% decrease in the ABTS antioxidant capacity of carrots after HPP at 500 MPa for 5 min (4°C). In another study, a decrease in ABTS antioxidant capacity of orange juice was observed when the HPP pressure level was increased from 100 to 800 MPa (at 30–65°C) (60) due to ascorbic acid degradation. In contrast, the DPPH antioxidant capacity of tomato puree was unchanged by HPP at 400 MPa/25°C/15 min (49) and Briones-Labarca et al. (44) reported an increase in the DPPH antioxidant activity of uncooked algarrobo seeds, an underutilized legume after high pressure processing at 500 MPA for 10 min compared to untreated seeds and seeds treated at 500 MPa for 2, 4 and 8 min (20°C). The reduced antioxidant activity recorded in the present study may have occurred because of the combination of cooking and high pressure processing resulting in destruction of the bioactive components or formation of new compounds with pro-oxidant action.

The correlation between total polyphenol content (TPC) and the antioxidant activity revealed by three assays (DPPH, ABTS and ORAC) is also represented in Table 5. A very high and significant (*P* < 0.001) positive correlation was observed between TPC and DPPH values (*r* = 0.922), and slightly lower but significant (*P* < 0.001) positive correlations with ABTS (*r* = 0.669), and ORAC (*r* = 0.727) assays were also observed. These results indicate that changes in the antioxidant capacity due to high pressure processing were closely related to the polyphenol content of the samples, supporting previous reports (24, 61).

**TABLE 5:** Pearson’s correlation between TPC, DPPH, ABTS, and ORAC values.

Parameters	DPPH	ABTS	ORAC
TPC	0.922*	0.669*	0.727*
DPPH		0.618*	0.703*
ABTS			0.655*

*Significant (p < 0.001). TPC, total polyphenol content; DPPH, 2-2-diphenyl-1-picrylhydrazyl antioxidant assay; ABTS, 3-ethylbenzothiazoline-6-sulfonic acid diammonium salt antioxidant assay; ORAC, oxygen radical antioxidant capacity.

### Scanning Electron Microscopy

**FIGURE 4:**
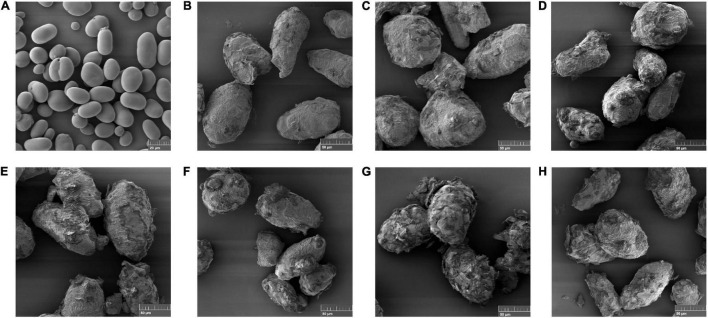
Scanning electron microscopy images (scale 20–50 μm) of isolated native starch (scale = 20 μm) **(A)**, control **(B)**, and high pressure processed cooked chickpeas (where, **(C)** = 200 MPa, 1 min; **(D)** = 200 MPa, 5 min; **(E)** = 400 MPa, 1 min; **(F)** = 400 MPa, 5 min; **(G)** = 600 MPa, 1 min; and **(H)** = s 600 MPa, 5 min).

Morphological characteristics of starch granules from raw, cooked and CHPP kabuli chickpeas was observed using scanning electron microscope (SEM). Raw chickpea starch (Figure 4A), which was used as an internal control exhibited smooth, oval shaped granules with no evidence of fissures or damage. Similar observations have been reported in previous studies on chickpea starch and starches from other legumes such as adzuki, black, and kidney bean (62, 63). When compared to starch granules from the raw samples, starch granules from the cooked and cooked+ HPP sample were a lot bigger (Figures 4A–H) in response to soaking and subsequent cooking. Scanning electron microscopy confirmed that high pressure processing altered the starch granule structure (Figures 4C–H). SEM pictures showed that, like the control (b), the majority of starch granule retain their granular shape. However, differences in their surface morphology was evident. HPP resulted in formation of fissures and caused surface damage to the starch granules of HPP samples. Similar results have been reported in previous studies on high pressure processing of barley (64) and potato (65) starch.

During high pressure treatment, the available water is forced into the chickpea grains causing rapid hydration driven by the applied external pressure, resulting in rapid swelling even at ambient temperatures (16). Błaszczak et al. (65) reported significant deformations to Freeze-dried potato starch granules caused by high pressure processing (65), which can be observed samples from this study as well. Liu et al. (66) reported that high pressure contributed to a strong interaction between amylose and amylopectin chains leading to formation of fractures and fissures on the surface of starch granules. This may also indicate significant changes in the internal structure of the granule. Starch undergoes a structural collapse that causes an alteration of the granular shape due to the simultaneous diffusion of solvents and gelatinization of starch (67). Taken at the same magnification as the control, the pictures of starch granules (Figure 4G) clearly depicts that very high pressure of 600 MPa is responsible for greater destruction of the granule integrity leading to further damage. As differences in the morphology of the isolated starch granules was observed, an *in vitro* starch digestibility assay was performed to understand the effect of HPP on starch digestibility of all samples.

### Attenuated Total Reflectance Fourier Transform Infrared Spectra Analysis

ATR-FTIR spectroscopy has previously been used to investigate the crystallinity, structure, and intermolecular interactions of starch granules (68). However, in previous studies using purified starch samples specific structural information relating to starch was analyzed (69, 70). In this study, control and HPP chickpeas have been analyzed with ATR-FTIR, and the spectra therefore contain contributions from proteins and lipids, in addition to starch. In this study, it is therefore not possible to draw absolute conclusions concerning starch structure, due to spectra overlap of starch absorbance bands with the absorbance bands of proteins and lipids.

**FIGURE 5:**
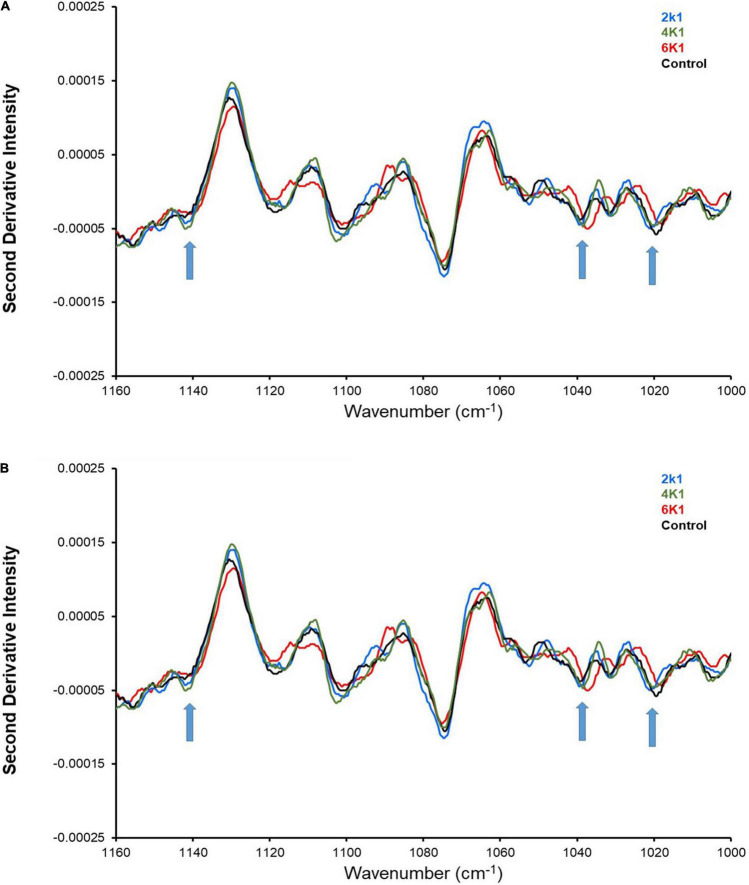
ATR-FTIR analysis of control and HPP kabuli chickpeas with upward arrows indicating shift in spectra at 1,140, 1,040, and 1,020 cm^– 1^ (where 2K1 = 200 MPa, 1 min; 2K5 = 200 MPa, 5 min; 4K1 = 400 MPa, 1 min; 4K5 = 400 MPa, 5 min; 6k1 = 600 MPa, 1 min; and 6K5 = 600 MPa, 5 min).

Nonetheless, ATR-FTIR is still a useful method to investigate relative changes in the starch bonding environment and/or structure, as a consequence of high pressure treatments at different times. The major adsorption bands arising from starch can be observed in the region 1,200–1,000 cm^–1^ and it has been shown that bands at 1,047 and 1,022 cm^–1^, respectively, describe the crystalline and amorphous indices of starch (71). The ATR-FTIR results (Figure 5) reveal distinct absorbance bands attributed to starch, which are easily viewed as “negative peaks” in the second-derivative spectra (Figures 5A,B). Three specific bands of interest are those at 1,140, 1,040, and 1,020 cm^–1^ [assigned to ν(C–O) modes] (68). Interestingly, an increased spectra shift to lower wavenumbers was observed at the bands for HPP samples treated at 200, 400 and 600 MPa, indicating treatment-induced alteration to the starch structure and intermolecular bonding environment.

Also, the intensity of the bands increased with an increase in treatment pressure and time (Figure 5B), suggesting conversion of starch molecules to its ruptured form and disruption of the chemical bonds (72). High pressure processing has been known to decrease the relative crystallinity of starch granules, which in turn lowers starch digestibility (73, 74). Pressurization during high pressure processing partially gelatinizes starch granules, whereas depressurization promotes retrogradation which results in recrystallization of amylopectin and further reduces starch digestion (75).

### *In vitro* Starch Digestibility

A number of factors such as source of starch, granule size, amylose-amylopectin ratio and the crystalline structure affect the enzymatic susceptibility of starches, with amylose content and the crystallinity being the most important factors (76). Starch gelatinization occurs when starch is heated in the presence of excess water, resulting in a change from crystalline to a more amorphous structure (76), thus becoming more digestible. However, starch entrapped within the cells of edible plant material (e.g., seeds) undergoes limited starch gelatinization (77), retaining its crystalline nature and resulting in a lower digestibility (78). Intracellular components such as proteins also impose restrictions on starch granule swelling, thus affecting starch digestibility (79).

**FIGURE 6:**
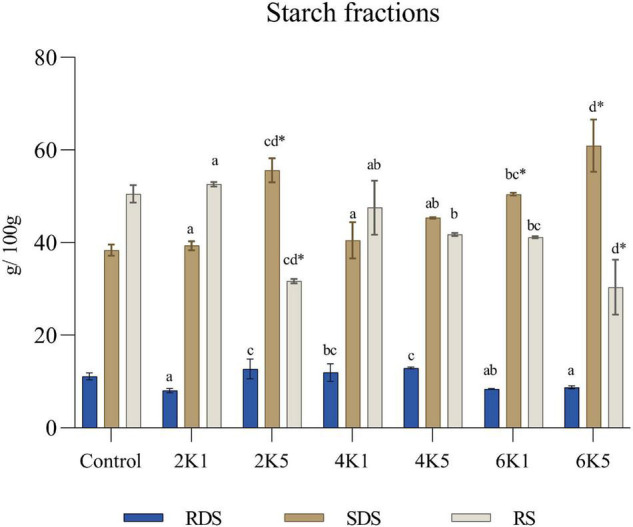
Rapidly digestible starch (RDS), slowly digestible starch (SDS) and resistant starch (RS) in the control and HPP chickpea samples. Bars bearing different letter for the same fraction are significantly (*p* < 0.05) different. Bars bearing * for the same fraction are significantly (*p* < 0.05) different than the control (where, 2K1 = 200 MPa, 1 min; 2K5 = 200 MPa, 5 min; 4K1 = 400 MPa, 1 min; 4K5 = 400 MPa, 5 min; 6k1 = 600 MPa, 1 min; and, 6K5 = 600 MPa, 5 min).

As shown in Figure 6, with an increase in treatment pressure and time, the contents of rapidly digestible starch (RDS), slowly digestible starch (SDS), and resistant starch (RS) in the HPP samples also changed. Significant (*P* < 0.05) main effects of pressure (P) and time (T) on RDS, SDS and RS levels were observed. It was observed that HPP at higher pressures and longer times (2K5, 4K5, and 6K5) can significantly (*P* < 0.05) increase the SDS levels in cooked chickpeas when compared to lower pressures and shorter treatment durations (2K1, 4K1, and 6K1), and when the pressure level reached the maximum value (600 MPa, 5 min) of the machine, the SDS fraction was at the highest amount (60.92 g/100 g starch). However, only the SDS content of the 2K5, 6K1, and 6K5 samples and the RS content of the 2K5 and 6K5 samples were significantly (*p* < 0.05) different from the control following the starch fractions determination.

Previous research has shown that high pressure processing can completely damage uncooked rice starch granules as well (80), while also promoting a wider distribution of water molecules in the crystalline regions of the starch granules. This in turn disrupts the amylopectin chains (81) and forms imperfect crystallites, which are directly linked to high SDS levels (82). Another reason for the increase in SDS content might be the smaller volume of amylose-lipid complexes formed in starch granules as a result of HPP (83).

Starch digestibility of cooked, waxy wheat starch (84), buckwheat (66), and brown rice (85) has previously been modified by the use of high pressure treatment, when compared to their raw counterparts. It was also observed in these studies that HPP increased the amount of slowly digestible starch (SDS), decreasing the overall starch digestibility of these samples. Slowly digestible starch has been shown to provide nutritional benefits for humans (86), as it leads to a slow and prolonged postprandial release of glucose in the blood stream, thus providing a lower glycemic response (87). It has been suggested that HPP has potential in producing products for glycemic control (85), which can help in the prevention of diseases such as diabetes, cardiovascular diseases and colon cancer (88).

### *In vitro* Protein Digestibility

The percentage *in vitro* protein digestibility (IVPD) values for the control and the highest HPP treatment (600 MPa, 5 min) are reported in Table 4. There was no significant (*P* < 0.05) effect of pressure, time or their interaction on the IVPD values for control and HPP sample. Cooking and thermal heat treatments (autoclaving) have been shown to improve the protein digestibility of raw legumes such as black grams, chickpeas, lentils, and red kidney beans due to an increased accessibility of the protein component for enzymatic attack (89), and/or inactivation of proteinacious anti-nutritional factors (90). High pressure cooking of rice has been shown to reduce its protein digestibility (91), whereas Laguna et al. (92) reported a small reduction (8%) in the protein digestibility of apple puree enriched with pea protein following HPP, with no effect of HPP on carrot puree enriched with pea protein. However, the effect of high pressure processing on the protein digestibility of chickpeas is not widely known.

Deol and Bains (93) reported an increase in protein digestibility of cowpeas after steam pressure cooking primarily due to destruction of antinutritional factors. Linsberger-Martin et al. (9) reported a 3.5% increase in the protein digestibility of split peas and a 6% increase in the protein digestibility of whole white beans after HPP at 600 MPa for 60 min. However, a small but significant reduction in protein digestibility was also reported in both split peas and whole white beans after HPP at 100 MPA for 60 min and 350 MPA for 45 min (9). A possible explanation for this reduction in protein digestibility can be due to the formation of intra/intermolecular disulfide bridges or a protein network (94). While the mechanism of pressure induced protein unfolding is not completely understood, the underlying mechanism of pressure induced protein denaturation involves water penetration into cavities within the molecule resulting in varying populations of molecular conformations (95), resulting in increased protein digestibility. However, as in our study all the samples were highly saturated with water (12 h soaking + 30 min boiling) before the HP treatment, no significant changes in the protein digestibility of control and HPP sample was observed. As there was no significant difference between the control and the highest treatment level (6K5), the remaining samples were not subjected to the analysis.

## Conclusion

This study demonstrated that the textural and starch digestibility properties of cooked kabuli chickpea is significantly improved by the application of high pressure processing. Significant effects of treatment pressure and time on the starch, polyphenol and antioxidant capacity were also revealed. These combined findings highlight the potential of utilizing non-thermal processing technologies to increase consumer acceptance of plant- based protein sources with desirable textural properties. Our findings suggest that high pressure processing has the potential to enable the design of products with a low glycemic index, compared to traditional products, while retaining dietary fiber percentages, minerals and digestible proteins and thus providing beneficial health effects to consumers. However, the shelf stability of cooked + high pressure processed ready-to eat chickpea product is unknown and thus requires further research.

## Data Availability Statement

The original contributions presented in the study are included in the article/supplementary material, further inquiries can be directed to the corresponding author.

## Author Contributions

PC, SJ, SB, and RC finalized the experimental design. PC carried out all the experiments and prepared the manuscript. SB, SJ, RC, and RB revised the prepared manuscript, contributed helpful discussion, and scientific advice during preparation of manuscript. All authors contributed to the manuscript and approved the submitted version.

## Conflict of Interest

SJ was employed at Ingredients by Design Pty Ltd. The remaining authors declare that the research was conducted in the absence of any commercial or financial relationships that could be construed as a potential conflict of interest.

## Publisher’s Note

All claims expressed in this article are solely those of the authors and do not necessarily represent those of their affiliated organizations, or those of the publisher, the editors and the reviewers. Any product that may be evaluated in this article, or claim that may be made by its manufacturer, is not guaranteed or endorsed by the publisher.
